# Isolated cecal necrosis mimicking acute appendicitis: a case series

**DOI:** 10.4076/1752-1947-3-7443

**Published:** 2009-06-19

**Authors:** Abuzer Dirican, Bulent Unal, Nuray Bassulu, Faik Tatlı, Cemalettin Aydin, Cuneyt Kayaalp

**Affiliations:** 1 Department of General Surgery, Inonu University School of Medicine, Malatya, Turkey; 2 Department of Pathology, Inonu University School of Medicine, Malatya, Turkey

## Abstract

**Introduction:**

Spontaneous non-occlusive ischemic colitis involving the cecum alone (isolated cecal necrosis) is a rare condition that is generally due to a low-flow state: shock. It presents with right lower quadrant abdominal pain and may resemble acute appendicitis. Little is known about postoperative ischemic necrosis of the remaining colon after surgical treatment of isolated cecal necrosis. We report four cases of isolated cecal necrosis mimicking acute appendicitis seen at our institution within a 4-year period.

**Case presentation:**

The patients were two men and two women with a mean age at diagnosis of 59 years (range 46-68). The patients were of Turkish ethnic origin. All patients presented to the emergency room with acute abdominal pain and had symptoms of nausea and vomiting. The medical histories for three of the patients revealed end-stage renal failure requiring hemodialysis. The other patient had chronic obstructive pulmonary disease. Right hemicolectomy with anastomosis was performed in three patients and cecal resection with ileocolostomy was performed in the remaining one patient. All of the patients healed without complications. Median follow-up of patients was 24.5 (range: 17-37) months. There was no recurrence of ischemia in the remaining colon during the follow-up period of the patients.

**Conclusion:**

Isolated cecal infarction should be included in the differential diagnosis of acute pain in the right lower quadrant of the abdomen, especially in those patients on chronic hemodialysis. While diffuse ischemic disease of the intestine has high morbidity, mortality and recurrence rates, patients with isolated cecal necrosis have a good prognosis with early diagnosis and surgical treatment compared to those with diffuse ischemic disease.

## Introduction

Ischemic colitis usually results from atherosclerosis and low blood flow. Acute colonic ischemia is a common cause of colitis in the elderly, in whom colonic ischemia is a cause of morbidity. However, ischemic colitis involving the cecum alone is quite rare, with only a few case reports in the literature. Isolated ischemic involvement of the right colon has been reported with increasing frequency, particularly in association with shock [1,2]. Cecal infarction presents with right lower quadrant pain, and therefore may resemble acute appendicitis. As this variant of ischemic colitis is less common, it may not be considered in the differential diagnosis of right lower quadrant pain. Given the possibility of cecal perforation, an early diagnosis and surgical treatment are very important factors in such cases. Little is known about postoperative ischemic necrosis of the remaining colon after surgical treatment of isolated cecal necrosis. Here, we report four patients with isolated cecal necrosis mimicking acute appendicitis seen at our institution within a 4-year period.

## Case presentation

This series is a retrospective analysis of isolated cecal necrosis cases diagnosed and treated at Inonu University Turgut Ozal Medical Center between July 2004 and June 2008. After a detailed examination of the medical records, four patients with confirmed diagnoses of isolated cecal necrosis were identified. Data regarding their age, sex, clinical presentation, comorbid diseases, imaging findings, treatment modality, and follow-up were reviewed. The results of hematological and biochemical analyses were evaluated, including the hemoglobin level, white blood cell count, lactate dehydrogenase, and alkaline phosphatase, as well as plain X-rays, abdominal ultrasound (US), and computed tomography (CT) of the abdomen.

The patients consisted of two men and two women with a mean age at diagnosis of 59 years (range 46-68). The patients were Turkish ethnic origin. All patients presented at the emergency room with acute abdominal pain and had symptoms of abdominal pain, nausea, and vomiting. Acute appendicitis was considered in all of the patients pre-operatively. The medical histories of three patients revealed end-stage renal failure requiring hemodialysis. The other patient had chronic obstructive pulmonary disease. The patients' laboratory findings and clinical characteristics are shown in Table 1.

**Table 1:** Patient characteristics and laboratory findings

Age, sex	Comorbid diseases	WBC	Hb (g/dL)	LDH (U/L)	CK (U/L)	Treatment	Follow-up (months)
58, F	CRI, DM, HT	23,700	11.1	618	238	Right hemicolectomy	17
68, F	CRI	16,400	16.5	323	647	Right hemicolectomy	19
64, M	COPD	19,400	12.1	911	50	Right hemicolectomy	25
46, M	CRI	21,000	13.6	262	719	Cecum resection	37

CRI, chronic renal insufficiency; DM, diabetes mellitus; HT, hypertension; COPD, chronic obstructive pulmonary disease; WBC, white blood cell count; Hb, hemoglobin; LDH, lactate dehydrogenase; CK, Creatine Kinase.

**Figure 1:**
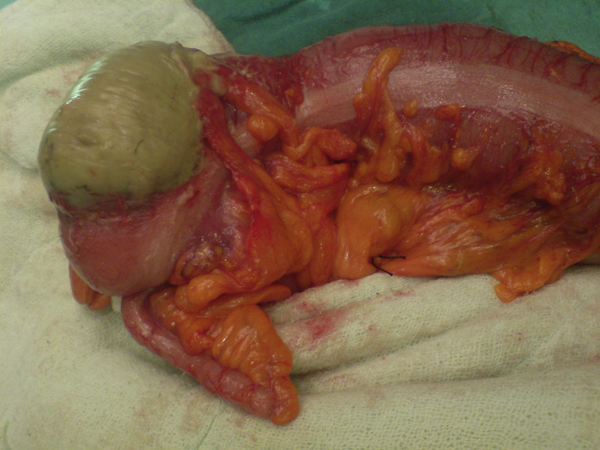
**Isolated cecal necrosis and a normal appendix**.

**Figure 2:**
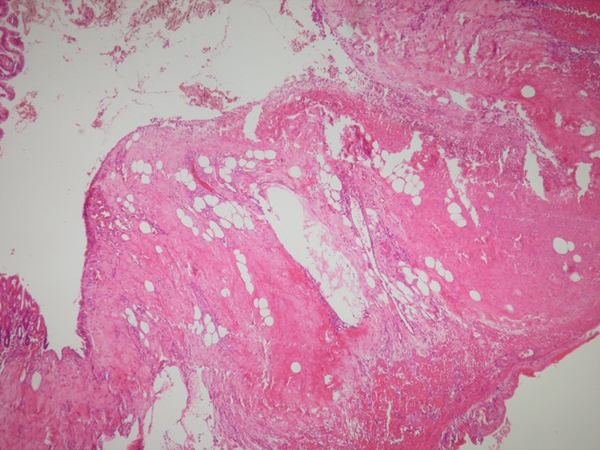
**Histopathological examinations revealed acute ischemic changes with transmural necrosis**.

There were no specific signs in the patients' pre-operative plain X-rays. Abdominal US revealed free intra-abdominal fluid in one patient, while the results of abdominal US were normal in the other patients. Only one patient underwent CT, the results of which were normal. In one patient, a diagnosis of cecal necrosis was made at diagnostic laparoscopy. At laparotomy, isolated cecal necrosis and a normal appendix were seen in all patients (Figure 1). Three patients were treated with right hemicolectomy, and the remaining one patient was treated with cecum resection. Histopathological examinations revealed acute ischemic changes with transmural necrosis and serositis isolated in the cecum in all patients (Figure 2). None of the patients showed mesenteric vascular occlusion. There were no postoperative complications, and the median follow-up period was 24.5 (range: 17-37) months.

## Discussion

Colonic ischemia is an significant cause of morbidity in the elderly. The causes of colonic ischemia can be classified as occlusive and non-occlusive [3]. Occlusive factors include atherosclerosis, thromboembolization, venous occlusion, and mechanical bowel obstruction. Non-occlusive colonic ischemia is due to a low-flow state (shock), which causes mesenteric vasoconstriction [1]. Isolated cecal necrosis can follow atherosclerotic or thromboembolic occlusion of the cecal arteries. Non-occlusive cecal infarction has been reported to occur in association with open-heart surgery, chronic heart disease, certain drugs, and hemodialysis [4]-[7].

Hemodialysis patients are at increased risk of ischemic colitis because they have accelerated rates of arterial vascular disease [8]. In addition, hypotension is common during dialysis as large amounts of fluid are removed during the procedure. Our three patients with end-stage renal disease underwent dialysis 3-4 times per week. We evaluated the patients' blood pressure during dialysis before the development of cecal necrosis, and a decrease to 60/40 was observed.

One patient had chronic obstructive pulmonary disease (COPD). There have been no previous reports of a connection between COPD and cecal necrosis. In this patient, the histopathology was reported as hemorrhagic ischemic necrosis, with patent mesenteric arteries. The patient may have had an insufficient blood oxygen level. Overall, isolated right colon or cecal necrosis is poorly understood [9].

Patients with this condition present with sudden-onset predominantly colicky, right-sided lower abdominal pain. An early clinical sign is pain or pressure in the right lower abdomen, which develops to general abdominal tenderness with muscular guarding within a few to 48 hours [8]. The symptoms in three of our patients began 24 hours after hemodialysis. All of the patients developed abdominal tenderness, guarding, and rebound tenderness. The initial symptoms were abdominal pain and nausea.

Isolated cecal necrosis may present a diagnostic challenge, as it is an unusual, less well-known, and rather atypical presentation of acute colonic ischemia [10]. Diagnosis is difficult because patients present with right-sided abdominal pain and tenderness, suggesting appendicitis, cecal diverticulitis, stercoral perforation, or cecal carcinoma. At present, there is no specific serum marker for colonic ischemia. Diagnostic US has been reported to be helpful in such cases [11]. CT shows nonspecific findings [12], although cecal wall thickening with isolated pneumatosis coli are strongly suggestive of the diagnosis [13]. Abdominal US was performed in all of our patients, while one patient underwent abdominal CT. There were no specific signs of cecal necrosis. To make a correct diagnosis, suspicion of cecal necrosis based on the clinical history and examination is very important. The use of colonoscopy in patients with suspected ischemic colitis is controversial. Bradbury et al. cautioned that colonoscopy may increase colonic perfusion because of increased transmural pressure [14]. We did not perform colonoscopy in any of our patients.

Diagnostic laparoscopy may be useful for diagnosis and treatment. Based on the results of diagnostic laparoscopy, we can choose the appropriate incision type. In one of our patients, the diagnosis of cecal necrosis was made at diagnostic laparoscopy, and a middle abdominal incision was chosen. Partial cecal necrosis can be treated by laparoscopic partial cecal resection [15]. Open surgery was chosen for our patient because of technical problems with the laparoscopic approach at the time of surgery.

If the clinical history and examination lead to suspicion of cecal necrosis, laparotomy should be performed without delay. A middle abdominal incision should be made to allow exploration of all of the intra-abdominal organs and intestine.

The treatment of choice for isolated cecal necrosis is cecal resection or right hemicolectomy. If evidence of peritonitis persists, right hemicolectomy with anastomosis can be performed satisfactorily [16]. One of our patients had 1000 mL of purulent fluid in the abdominal cavity. Cecal resection with an ileocolostomy was performed in this patient, and the patient healed without complications. Right hemicolectomy with anastomosis was performed in the other three patients. The appendix was normal in all patients.

Little information is available regarding the incidence of postoperative ischemic necrosis of the remaining colon after surgical treatment of isolated cecal necrosis. The postoperative course in all of our patients was uneventful. There was no new intestinal vascular occlusion or ischemic colitis in the postoperative period.

## Conclusion

Isolated cecal infarction should be included in the differential diagnosis of acute right lower quadrant pain, especially in chronic dialysis patients. While ischemic diseases of the intestine have high morbidity and mortality rates, with early diagnosis and surgery, isolated cecal necrosis has a good prognosis.

## Consent

Written informed consent was obtained from the patients before publication of this case series and any accompanying images. A copy of the written consent is available for review by the Editor-in-Chief of this journal.

## Competing interests

The authors declare that they have no competing interests.

## Authors' contributions

AD is the consultant surgeon who drafted the article and performed the operations. BU assisted in performing the surgery, took the pictures and helped revise the article. NB made the histopathologic diagnosis of the patients. CA and FT helped in surgical treatment of the patients. CK helped in acquisition of data and technical support. All authors have read and approved the final manuscript.
